# Sustaining remission of psychotic depression: rationale, design and methodology of STOP-PD ΙΙ

**DOI:** 10.1186/1471-244X-13-38

**Published:** 2013-01-25

**Authors:** Alastair J Flint, Barnett S Meyers, Anthony J Rothschild, Ellen M Whyte, Benoit H Mulsant, Matthew V Rudorfer, Patricia Marino

**Affiliations:** 1 Department of Psychiatry, University of Toronto, Toronto, Canada; 2 Department of Psychiatry, University Health Network, Toronto, Canada; 3 Toronto General and Toronto Rehab Research Institutes, Toronto, Canada; 4 Department of Psychiatry Weill Medical College of Cornell University and New York Presbyterian Hospital–Westchester Division, New York, USA; 5 University of Massachusetts Medical School and UMass Memorial Health Care, Worcester, USA; 6 Western Psychiatric Institute and Clinic, Department of Psychiatry, University of Pittsburgh School of Medicine, Pittsburgh, USA; 7 Centre for Addiction and Mental Health, Toronto, Canada; 8 National Institute of Mental Health, Bethesda, USA; 9 Toronto General Hospital, 200 Elizabeth St., 8 Eaton North–Room 238, Toronto, Ontario, M5G 2C4, Canada

**Keywords:** Major depressive disorder with psychotic features, Psychotic depression, Randomized controlled trial, Antipsychotic discontinuation, Relapse, Metabolic effects, Aged, Multi-center study

## Abstract

**Background:**

Psychotic depression (PD) is a severe disabling disorder with considerable morbidity and mortality. Electroconvulsive therapy and pharmacotherapy are each efficacious in the treatment of PD. Expert guidelines recommend the combination of antidepressant and antipsychotic medications in the acute pharmacologic treatment of PD. However, little is known about the continuation treatment of PD. Of particular concern, it is not known whether antipsychotic medication needs to be continued once an episode of PD responds to pharmacotherapy. This issue has profound clinical importance. On the one hand, the unnecessary continuation of antipsychotic medication exposes a patient to adverse effects, such as weight gain and metabolic disturbance. On the other hand, premature discontinuation of antipsychotic medication has the potential risk of early relapse of a severe disorder.

**Methods/design:**

The primary goal of this multicenter randomized placebo-controlled trial is to assess the risks and benefits of continuing antipsychotic medication in persons with PD once the episode of depression has responded to treatment with an antidepressant and an antipsychotic. Secondary goals are to examine age and genetic polymorphisms as predictors or moderators of treatment variability, potentially leading to more personalized treatment of PD. Individuals aged 18-85 years with unipolar psychotic depression receive up to 12 weeks of open-label treatment with sertraline and olanzapine. Participants who achieve remission of psychosis and remission/near-remission of depressive symptoms continue with 8 weeks of open-label treatment to ensure stability of remission. Participants with stability of remission are then randomized to 36 weeks of double-blind treatment with either sertraline and olanzapine or sertraline and placebo. Relapse is the primary outcome. Metabolic changes are a secondary outcome.

**Discussion:**

This trial will provide clinicians with much-needed evidence to guide the continuation and maintenance treatment of one of the most disabling and lethal of psychiatric disorders.

**Trial registration and URL:**

NCT: NCT01427608

## Background

Major depression with psychotic features (psychotic depression; PD) is a severe, potentially fatal disorder with a high risk of relapse and recurrence [1,2]. Older adults are at greatest risk of PD, with up to 45% of older inpatients with major depression having psychotic features [3,4]. Electroconvulsive therapy (ECT) and pharmacotherapy are each efficacious in the treatment of PD [5,6]. Several factors influence the choice of treatment, including patient preference, clinical acuity, past history of treatment response, side effect profile, and availability of ECT.

When pharmacotherapy is selected, expert guidelines, supported by meta-analytic evidence, recommend a combination of antidepressant and antipsychotic medications for acute treatment [7-9]. However, little is known about the continuation and maintenance treatment of PD. Once an episode of major depression responds to antidepressant medication, the antidepressant needs to be continued to prevent relapse and recurrence of depression [10]. However, it is not known whether antipsychotic medication needs to be continued once an episode of PD has responded to combined antidepressant-antipsychotic treatment. This issue is of profound clinical importance: on the one hand, premature discontinuation of antipsychotic medication has the potential risk of relapse of a severe, life-threatening disorder; on the other hand, the unnecessary continuation of antipsychotic medication exposes a patient to potentially serious adverse effects.

There are few data on the relation between discontinuation of antipsychotic medication and relapse of PD. In a chart review of persons with PD who had responded to pharmacotherapy, Aronson et al. [11] found that approximately 35% of relapses occurred following the discontinuation of antipsychotic medication in persons who continued to take antidepressant medication. The only prospective study of antipsychotic discontinuation in PD found a frequency of relapse of 27% over 8 months in 30 mid-life adults who had recovered with combined fluoxetine and perphenazine [12]; the findings of this study are, however, limited by its open and uncontrolled design, the relatively small number of subjects, and the absence of older patients who, based on studies of non-PD, may conceivably be at greater risk of relapse [13]. In contrast to these discontinuation data, three small open-label studies suggest that patients with PD who are *maintained* on the treatment to which they responded may have only a 0-6% rate of relapse over 12 months [14-16].

The recently completed Study of the Pharmacotherapy of Psychotic Depression (STOP-PD) [17] was the first NIMH-funded randomized controlled trial (RCT) to examine the efficacy and tolerability of combination pharmacotherapy using a serotonergic antidepressant and a second generation antipsychotic medications in the acute treatment of PD. The combination of sertraline and olanzapine was significantly more efficacious than olanzapine monotherapy. The two treatments had comparable tolerability. Nevertheless, both treatments were associated with an increase in weight and lipids over the 12-week study. The primary goal of STOP-PD II (‘Sustaining Remission of Psychotic Depression’) is to assess the benefits and risks of continuing antipsychotic medication in younger and older patients with PD, once the episode of psychotic depression has responded to treatment with sertraline and olanzapine. This goal will be addressed through a 36-week double blind RCT, in which placebo is substituted for olanzapine in half the study group, following a period of sustained remission. The study provides the unique opportunity to systematically assess the effect of antipsychotic discontinuation on olanzapine-related weight gain and metabolic disturbance. Olanzapine was chosen because it is the only antipsychotic medication with established efficacy in combination therapy in both younger and older persons with psychotic depression. Additional aims of the study are to examine age and genetic polymorphisms as predictors/moderators of treatment variability. The purpose of this article is to describe and discuss selected aspects of the rationale, design, and methodology of STOP-PD II, in order to highlight research and clinical issues that are pertinent to the longer-term pharmacologic management of PD. The study has been approved by the local review boards of the four participating sites: University Health Network, Toronto; University of Massachusetts Medical School; University of Pittsburgh School of Medicine; and Weill Cornell Medical College. Quality assurance and safety issues are overseen by a Data and Safety Monitoring Board appointed by the National Institute of Mental Health.

## Methods/design

### Overview of design

The study has 3 phases: acute, stabilization, and randomized. We plan to enroll 392 patients (n = 196 aged 18-59 years and n = 196 aged ≥60 years) with Structured Clinical Interview for DSM-IV-TR [18] (SCID)-defined non-bipolar major depression with psychotic features (delusions, with or without hallucinations) across 4 sites into open-label treatment with the combination of sertraline (target dose of 150-200 mg/day) and olanzapine (target dose of 15-20 mg/day). Participants will continue with open-label sertraline and olanzapine for an 8-week stabilization phase if they no longer have delusions and hallucinations and either a) have a 17-item Hamilton Depression Rating Scale (HAM-D) [19] score of ≤10 for 2 consecutive weeks (‘full-remission’), or b) are rated ‘very much improved’ or ‘much improved’ on the Clinical Global Impression (CGI) Scale [20] and have a HAM-D score of 11-15 with ≥50% reduction in their baseline HAM-D score by the end of the acute phase (‘near-remission’). While the acute phase of the study can last a maximum of 12 weeks, participants enter the stabilization phase as soon as they meet full-remission criteria. Participants who continue to meet full-remission or near-remission criteria and have a Mini-Mental State Examination (MMSE) [21] score of ≥24 at the end of the stabilization phase enter the 36-week RCT. Participants are randomized under double-blind conditions to either continue olanzapine or switch from olanzapine to placebo following a 4-week placebo-controlled taper of the olanzapine. All participants take open-label sertraline for the duration of the RCT. Relapse is the primary outcome.

### Rationale for aspects of the design

#### Duration of acute treatment

Although most studies of the acute treatment of PD have been of 6-8 weeks duration [6,9], a maximum of 12 weeks of acute treatment is more suitable for this study for several reasons. First, one of the goals of the acute phase is to maximize the chance of achieving remission of delusions and antidepressant response, in order for participants to enter the RCT. In STOP-PD, one-third of participants who experienced remission with combined sertraline and olanzapine did not do so until between weeks 6 and 12 of treatment. Second, studies of 6-8 weeks typically use one cross-sectional assessment of ≥50% decrease in severity of depression as the primary outcome criterion. The acute treatment outcome criterion used in this study requires a longer period of observation. On the other hand, the decision to limit acute treatment to a maximum of 12 weeks is based on funding considerations and the fact that patients who do not experience remission of psychosis and at least substantial improvement in depression following 12 weeks of intensive antidepressant-antipsychotic treatment should probably be considered for additional or alternative treatment.

#### Duration of stabilization phase

There are no data, and there is no consensus among experts, about how long to continue antipsychotic medication, once an episode of PD has responded to combination pharmacotherapy. However, a survey of 50 experts in the treatment of late-life depression found that the majority would not recommend stopping antipsychotic medication immediately after remission of PD [22]. In the absence of data to the contrary, we believe that it is prudent to allow for a period of stability of remission of psychosis prior to withdrawal of the antipsychotic.

#### Discontinuation design

The primary aim of this study is to assess whether olanzapine, in combination with antidepressant medication, prevents relapse of PD. This question can be addressed through a RCT, in which placebo is substituted for olanzapine in half the study participants at a fixed point in time. We also considered a placebo-controlled sequential discontinuation design, whereby placebo is substituted for olanzapine at different time points. Whilst a sequential discontinuation design could provide additional information about how long to continue olanzapine once an episode of PD has remitted, it would require many more study participants than the design that we propose, and would therefore be more challenging in terms of recruitment and much more costly than the current study.

#### Duration of the RCT

Limited data from naturalistic studies of PD suggest that when antipsychotic medication is discontinued, the first 3 months after discontinuation is the period of greatest risk of relapse [11]. The choice of 36 weeks for the RCT therefore: a) covers the period of greatest risk of relapse of PD following the discontinuation of the antipsychotic, yet allows for a period of observation beyond that time, b) saves on the cost that would be associated with a longer study, and c) allows the study to be completed within 5 years in the four collaborating sites.

#### Duration of antipsychotic taper

In Rothschild et al’s [12] open-label study of the withdrawal of antipsychotic medication in PD, antipsychotic medication was tapered over a period of 4 weeks. No patient experienced discontinuation side effects or re-emergence of psychosis during the 4-week taper.

#### Stratification by Age

PD is prevalent in older adults with major depression [3,4,23]. Age-related pharmacodynamic and pharmacokinetic changes may result in age-related differences in treatment efficacy and tolerability [24]. It is therefore important to include a sufficient number of older persons in order to examine the relation between age, efficacy, tolerability, and safety.

#### Remission criteria to enter the RCT

Studies of major depression have found that experiencing only partial improvement in depressive symptoms may increase the risk of subsequent relapse of depression [25]. We therefore believe that, in addition to experiencing full remission of psychosis, participants should experience full remission or *substantial* improvement in their depressive symptoms prior to changes being made to the treatment to which they responded. Given that patients in this study continue with an antidepressant throughout the RCT, and may therefore continue to experience improvement in depression beyond the acute/stabilization phases of treatment, we include persons with substantial improvement in depressive symptoms, even though the symptoms may not have reached the conventional < 8-10 HAM-D cut point for remission used in studies of non-psychotic depression; this approach reflects ‘real world’ clinical practice.

#### MMSE score ≥24 to enter the RCT

Dementia can be associated with depressive and psychotic symptoms that respond differently to pharmacotherapy from major depressive disorder with psychotic features. As described in the Eligibility section below, persons with evidence of clinically significant cognitive decline preceding the index episode of PD, a marker for current or incipient dementia, are excluded from the study. In addition, any participant with a MMSE score <24 at the end of the stabilization phase is excluded from participation in the RCT, since poor cognitive performance at that stage of the study cannot be explained by depression or psychosis.

### Hypotheses and exploratory aims

#### Primary hypothesis

H1.The combination of sertraline and olanzapine will be associated with less risk of relapse than the combination of sertraline and placebo.

#### Secondary hypotheses

H2. The combination of sertraline and olanzapine will be associated with higher weight, higher total cholesterol, and higher triglycerides compared with the combination of sertraline and placebo in the randomized phase.

H3. Older age will be associated with less weight gain than younger age during the open-label phase.

#### Exploratory aims

i) Explore older age as a moderator of change in weight, lipids, and insulin resistance during the randomized phase. ii) Explore the association of selected genetic polymorphisms with: a) response, b) relapse, and c) weight, lipids, and insulin resistance during the open-label and randomized phases of the study.

### Recruitment and eligibility

Patients are recruited from inpatient units, outpatient programs, and affiliated community hospitals of the 4 academic healthcare centers that participated in STOP-PD (i.e., the medical centers of Cornell University, the University of Massachusetts, University of Pittsburgh, and University of Toronto). Inclusion/exclusion criteria are described in Table 1. Several of these criteria deserve specific mention. First, participants are required to have delusions, whether or not hallucinations are present. This increases diagnostic precision, since psychotic patients with hallucinations alone are more likely to be suffering from another disorder, such as schizophrenia, schizoaffective disorder, brief psychotic disorder, or a toxic-metabolic encephalopathy, as a cause of their psychotic symptoms [26]. Second, given that the validity of a study of psychotic depression rests on the presence of delusions (as opposed to other abnormal beliefs such as overvalued ideas, obsessions, or anxious worries) participants in STOP-PD II must score >2 on at least one of the three conviction items of the Delusion Assessment Scale (DAS) [27]; that is, they must be certain that the delusional belief is true and not modify the belief or consider alternative explanations in response to reality testing by the interviewer. Third, persons with dementia are excluded because delusions and depression may be symptomatic manifestations of dementia [28]. The pathophysiology, treatment response, and course of these symptoms of dementia may differ from those of PD. In addition, non-delusional confabulations are a frequent symptom of dementia and may be difficult to reliably distinguish from delusions [28]. Moreover, dementia may affect a person’s ability to reliably and accurately report the presence and course of symptoms, which could affect the validity of ratings of outcome. Finally, persons with Type 1 diabetes mellitus are at risk of ketoacidosis if they become hyperglycemic, whereas ketoacidosis is a very rare event in persons with Type 2 diabetes [29]. Therefore, to avoid the potential risk of ketoacidosis precipitated by olanzapine-associated hyperglycemia, persons with Type I diabetes are excluded from the study.

**Table 1:** STOP-PD II Inclusion and Exclusion criteria

**Inclusion criteria**	
1)	Aged 18-85 years, inclusive
2)	Diagnosis: DSM-IV non-bipolar major depression with psychotic features, established through both a clinical interview by a research psychiatrist and the subsequent administration of the SCID-IV by a research associate
3)	Score of ≥3 on the delusion severity item of the SADS (‘delusion definitely present’), with or without hallucinations on the SADS hallucination item
4)	Score of >2 on any of the three conviction items of the DAS (the participant is certain a belief is true and does not change the belief in response to reality testing by the interviewer);
5)	17-item Ham-D score of >21.
**Exclusion criteria**	
1)	Current or lifetime DSM-IV criteria for: schizophrenia, schizoaffective disorder or other psychotic disorder, mental retardation, or meeting DSM-IV criteria for current brief psychotic disorder, body dysmorphic disorder, or obsessive-compulsive disorder
2)	Current or lifetime DSM-IV criteria for bipolar affective disorder
3)	History of DSM-IV defined substance abuse or dependence, including alcohol, within the last three months
4)	DSM-IV defined Alzheimer’s dementia, vascular dementia, or dementia due to other medical conditions, or a history of clinically significant cognitive impairment prior to the index episode of depression, and/or a mean score of ≥4 on the 26-item IQCODE. The IQCODE will be used to screen for clinically significant cognitive decline that began prior to the index episode of PD (a cut score of 4 has been found to have a sensitivity of 84-93% and specificity of 88-94% in screening for dementia in general, psychiatric, and medical populations of older adults [30,31]
5)	Type 1 diabetes mellitus (defined as insulin-dependent diabetes mellitus with onset < 35 years of age and/or diabetes mellitus that has been complicated by a prior documented episode of ketoacidosis)
6)	Acute or unstable medical illnesses (e.g., delirium; metastatic cancer; unstable diabetes, decompensated cardiac, hepatic, renal or pulmonary disease; stroke; or myocardial infarction) within the last three months; current abnormal serum free T4; current abnormally low serum vitamin B12 or folic acid level; medical conditions and/or medications for which psychotic or depressive symptoms can be a direct manifestation (e.g. Cushing’s disease, high-dose systemic corticosteroids, L-dopa); neurological disease associated with extrapyramidal signs and symptoms (e.g. Parkinson’s disease); epilepsy, if the person has had one or more grand mal seizures in the past 12 months
7)	The need for treatment with any psychotropic medications other than sertraline, olanzapine, or lorazepam; or with an anticonvulsant medication with mood-stabilizing properties (carbamazepine, lamotrigine, valproic acid)
8)	Current pregnancy or a plan to become pregnant during the duration of the study in woman of childbearing age; breast-feeding in woman with infants
9)	A clearly documented history of being unable to tolerate sertraline and/or olanzapine, including having had an untoward previous reaction to sertraline such as significant bradycardia (heart rate of <50 bpm) or development of the syndrome of inappropriate antidiuretic hormone secretion with a serum sodium of 129 mmol/L or below
10)	History of non-response of the index episode of PD to at least a 6-week trial of ≥150 mg/day sertraline combined with ≥ 15 mg/day olanzapine
11)	Patients showing ongoing improvement in the index episode of PD with treatment, other than sertraline and olanzapine, initiated prior to the study
12)	Sufficiently ill to require immediate ECT (e.g., imminent risk of suicide, refusing to eat or severe malnutrition, catatonic)

### Informed consent

PD may impair a person’s capacity to consent to participate in a research study. To recruit people who are representative of the population being studied, the study allows people who do not have the capacity to give informed consent to participate in the open-label phase of the study (acute and stabilization). Capacity is determined by either an assessment conducted by a licensed mental healthcare professional who is independent of the study or by administration of the MacArthur Competence Assessment Tool for Clinical Research (MacCAT-CR) [32]. The MacCAT-CR is a semi-structured interview for assessment of capacities related to consent to research; it has been found to be a valid measure of capacity to consent in persons with psychosis. Individuals who lack capacity can participate if they assent to participate and informed consent is obtained from a surrogate decision maker. Subjects who are eligible for the randomized phase must have achieved sustained remission or near remission by the end of stabilization and therefore must be capable of giving their own informed consent to participate in the RCT.

### Treatment regimen

#### Acute and stabilization (open-label) phases

The only psychotropic medications allowed in the study are i) olanzapine, ii) sertraline, iii)‘as needed’ lorazepam or stable doses of other benzodiazepines if they were being taken prior to study entry and the participant is not able to cross-taper to lorazepam, and iv) ‘as needed’ benztropine. When participants meet remission criteria, they continue with open-label sertraline and olanzapine during the 8-week stabilization phase. Since the goal of the stabilization phase is to consolidate stability of remission, adjustment of the doses of study medications is allowed, if necessitated by clinical worsening or significant side effects. Because sustained remission of psychosis during the stabilization phase is required for eligibility for the RCT, participants leave this phase if they experience a relapse of delusions or hallucinations during this time as determined by SCID interview.

#### Randomized phase

Participants who continue to meet the study’s remission criteria at the end of the stabilization phase are randomly allocated to either continue olanzapine or switch from olanzapine to placebo. All participants take open-label sertraline for the duration of the RCT. The goal is to maintain sertraline at the same acute dose as the one prescribed at the time of randomization to the RCT. Because participants have taken sertraline for 12-20 weeks before entering the RCT, we anticipate that the dose of sertraline will rarely need to be adjusted in the RCT because of adverse effects. However, a reduction of the dosage of sertraline is allowed if the site PI agrees. In contrast, an increase in dose of sertraline is not permitted at any time during the RCT, because it will confound testing of the primary hypothesis. Similarly, the goal is to maintain olanzapine/placebo at the same acute dose as the one prescribed at the time of randomization to the RCT. However, a change in dose of olanzapine/placebo is permitted, if necessitated by adverse effects or clinical worsening during the RCT, following discussion with the site PI. The rationale for allowing a change in olanzapine/placebo dose is that the primary aim of the study is to assess whether olanzapine, administered within a clinically relevant dose range, prevents relapse, not whether a specific dose of olanzapine prevents relapse.

### Relapse criteria

Relapse is the primary outcome. Relapse criteria are broad, to reflect a range of clinically relevant outcomes of PD. Declaring relapse requires at least one of the following: 1) symptoms of DSM-IV major depression; 2) 17-item HAM-D score of ≥18; 3) re-emergence of SCID-rated psychosis (delusions or hallucinations) and a score of ≥3 on the Schedule of Affective Disorders and Schizophrenia (SADS) [33] delusion or hallucination severity items (delusion/hallucination ‘definitely present’); 4) other significant clinical worsening, defined as: i) suicide plan or suicide attempt at any time, ii) development of SCID-rated symptoms of mania or hypomania, or iii) psychiatric hospitalization for depression, psychosis, suicidality, or mania/hypomania, regardless of the duration of these symptoms. These relapse criteria reflect a clinical deterioration for which most clinicians would consider a change of treatment. Even though patients with a history of bipolar disorder will not be allowed to enter the study, mania or hypomania is included as an outcome variable because onset of PD in younger adults may predict subsequent development of bipolar disorder [1,2]. Participants with relapse will leave the study and be treated under usual care conditions.

### Sample estimates

**Figure 1:**
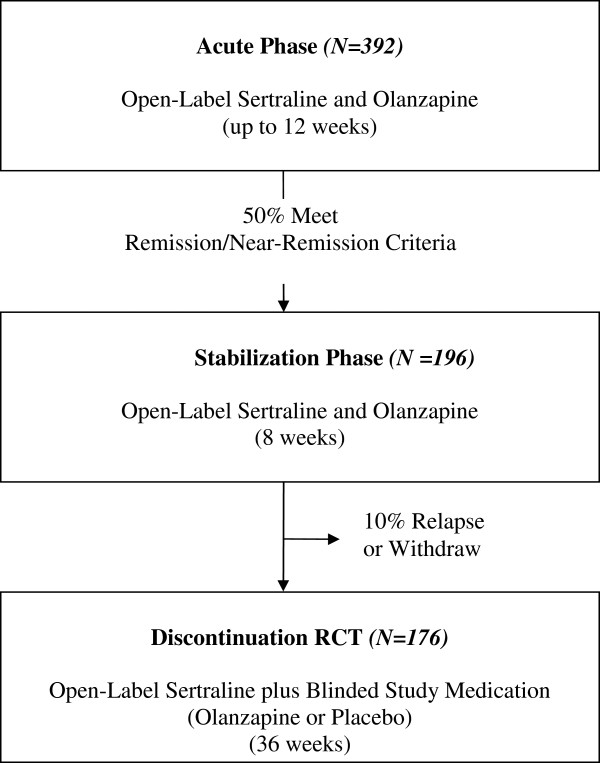
STOP-PD II: study design and subject flow.

Of the 392 subjects recruited to the acute phase of the study, we anticipate that 196 (50%) will achieve remission or near-remission as defined above. Based on STOP-PD pilot stabilization data and findings of the study by Rothschild et al. [12], we estimate that approximately 10% of acute phase full/near-remitters will not complete the 8-week stabilization phase, either because of relapse or discontinuation. Thus, we predict that 176 participants will be randomized (Figure 1).

### Measures and schedule of study visits

#### Primary clinical measures

Pertain to the study’s aims and hypotheses or eligibility criteria. They are the SCID-IV, GRID version of the 17-item HAM-D [34], DAS, delusion and hallucination items of the SADS, Scale for Suicidal Ideation (SSI) [35], CGI, IQCODE [36], and MMSE (Table 2).

**Table 2:** STOP-PD II schedule of events: acute and stabilization phases and randomized controlled trial (RCT)

**Instrument**	**Baseline**	**Acute phase**^**a**^**(week) (4-12 wks in duration)**			**Stabilization phase (week)**		**Discontinuation RCT (week)**														
		**4**	**8**	**12**^**b**^	**4**	**8**	**1**	**2**	**3**	**4**	**5**	**6**	**7**	**8**	**12**	**16**	**20**	**24**	**28**	**32**	**36**^**c**^
SCID	x																				x
Clinical Ratings^d^	x	x	x	x	x	x	x	x	x	x	x	x	x	x	x	x	x	x	x	x	x
Weight & Waist Circumference	x	x	x	x	x	x	x	x	x	x	x	x	x	x	x	x	x	x	x	x	x
Height	x																				
Metabolic Labs	x	x		x		x								x		x		x		x	x
Drug Plasma Levels		x		x		x										x					x
Genetic Testing	x																				
Baseline Physical^e^	x																				
Vital Signs	x	x	x	x	x	x	x	x	x	x	x	x	x	x	x	x	x	x	x	x	x
Anxiety^f^	x					x															
Medical Burden^f^	x					x															
Psychomotor Change^f^	x					x															
Cognition^g^	x					x															
Treatment Resistance^h^	x																				
Quality of Life^i^	x					x															x
EPSE Ratings	x	x	x	x	x	x				x				x	x	x	x	x	x	x	x
Best Guess Form^j^																					x
Pill Count		x	x	x	x	x	x	x	x	x	x	x	x	x	x	x	x	x	x	x	x

^**a**^ The duration of the Acute Phase is variable and can last between 4 and 12 weeks, depending on time to response.

^**b**^ These assessments are completed at the end of the Acute Phase, which occurs between 4 and 12 weeks after starting study medications.

^**c**^ These assessments are completed at week 36 or at the point of termination.

^**d**^ Primary clinical ratings are HAM-D, DAS, SADS delusion and hallucination items, SSI, and CGI. In addition, the UKU side effects scale, Falls Log, and Concomitant Medication Log will be completed at these visits.

^**e**^ Baseline physical includes physical examination, screening blood tests (CBC, electrolytes, creatinine, AST, ALT, TSH, Vitamin B12, pregnancy test for premenopausal women), urine drug screen, and EKG.

^f^ Measures of anxiety, medical burden, and psychomotor change are the Hospital Anxiety and Depression Scale [37], Cumulative Illness Rating Scale [38], and CORE [39], respectively.

^**g**^ Cognitive evaluation includes: the IQCODE at baseline; and the MMSE, Immediate and Delayed Recall, Stroop, Trail Making, and Coding task [40,41] at the end of the Stabilization Phase.

^h^ Treatment resistance during the index episode of depression is measured with the Antidepressant Treatment History Form [42].

^i^ The SF-36 [43] will be administered to: i) examine the association of acute and remitted psychotic depression with participants’ health-related quality of life, and ii) explore the effect of continuation versus discontinuation of antipsychotic medication on participants’ health-related quality of life.

^j^ The Best Guess Form is completed by participants at RCT termination, to determine, once the study is completed, whether they can guess treatment assignment on a greater than chance basis; this will serve as an indicator of how well the study’s double-blind was preserved.

#### Secondary clinical measures

Anxiety symptoms, medical burden, executive dysfunction, psychomotor change, and ‘treatment resistance’ during an index episode of depression have each been associated with increased risk of relapse/recurrence of major depression [44-48]. These variables are therefore included as covariates in the Cox model examining risk of relapse. In addition, given the age range of study participants, selected cognitive tests (delayed recall, psychomotor speed, and cognitive flexibility) that target previously described impairments in PD [49] and late-life depression [50] are administered at entry in to the RCT to help characterize the RCT sample.

#### Measures of Non-metabolic safety

Are the UKU Side Effects Scale [51], Simpson Angus Scale [52] to measure drug-associated parkinsonism, Barnes Akathisia Scale [53], Abnormal Involuntary Movement Scale [54] to measure tardive dyskinesia, orthostatic hypotension, and frequency of falls.

#### Anthropometric measures

The selection of anthropometric measures and metabolic parameters is based on consensus recommendations regarding the clinical monitoring of patients treated with atypical antipsychotics [55]. Body mass index (BMI) and waist circumference are key clinical measures of body fat [56]: BMI is an index of total body fat, whereas waist circumference is a measure of abdominal obesity, which is more specifically associated with cardiovascular risk and metabolic syndrome than BMI. BMI and waist circumference are assessed at each study visit.

Analyses of antipsychotic weight gain in major depression can be confounded by depression-related weight loss and weight restoration associated with recovery from depression. Therefore, participants’ pre-morbid weight is obtained, preferably from the patient’s primary care physician’s records. Pre-morbid weight will be used to calculate weight loss and weight restoration during the index episode of depression, which will be taken in to account in statistical analyses of weight change.

#### Metabolic measures

Homeostatic model assessment (HOMA) [57], a measure of insulin resistance and the primary measure of glycemic function in this study, will be calculated based on an established formula, each time fasting glucose and insulin are measured. HbA1c is a secondary measure of glycemic function, to assess the longitudinal stability of serum glucose levels during the course of the study. Triglycerides and cholesterol (total, LDL, HDL) are also measured.

#### Drug exposure

Pharmacokinetic changes associated with aging and other individual factors can result in variability of drug exposure, which in turn can result in differences in pharmacodynamics [24]. Drug exposure is therefore an important piece of information when interpreting variability in treatment efficacy and adverse effects [58]. Blood is collected for determination of plasma sertraline and olanzapine concentrations. The analytic strategy will use population pharmacokinetics [59], which uses nonlinear mixed effect modeling to identify intra- and inter-individual sources of variability [60]. Variability from the norm in drug concentrations can be determined using sparse (between two and four) plasma samples per patient [61].

#### Pharmacogenetics

As with other psychiatric disorders, the pharmacologic treatment of PD is associated with considerable treatment variability. Pharmacogenetic findings have the potential to result in more personalized treatment and possibly preventive strategies. Because the number of individuals recruited in a clinical trial is insufficient to conduct a genome-wide association study, we employ a candidate-gene approach to explore the following important issues: 1) genetic predictors of relapse of either mood disorders or primary psychotic disorders are currently unknown: we therefore explore this issue in relation to this study; 2) several genetic polymorphisms have been associated with antipsychotic-related weight gain [62]. However, it is not known whether these polymorphisms predict reversal of weight gain following discontinuation of the antipsychotic, an issue that we will investigate; 3) there have been no published studies of the pharmacogenetics of response or remission in PD. We will therefore explore whether genetic polymorphisms that have been associated with antidepressant response in non-psychotic depression, and antipsychotic response and weight gain in schizophrenia, pertain to PD. Finally, we participate in the National Institute of Mental Health’s Human Genetics Initiative so that cell lines, DNA, and genetic data from our very well characterized sample will be available to the wider scientific community. For example, there is emerging evidence that certain genes may confer risk for severe psychiatric illness, as opposed to specific disorders [63,64]. The genetic data from our study will provide scientists with the unique opportunity to include persons with PD in analyses pertaining to these ‘susceptibility genes’.

### Data analysis

#### Randomization

We plan to randomize 88 participants to each of the two treatment arms using a 1:1 allocation ratio, stratified by age, remission vs. near remission status at randomization, and site. In order to reduce the probability that a disproportionate number of subjects are randomized to any one level of the factors, a blocking strategy will be used.

#### Primary hypothesis (H1)

This will be tested with a Cox proportional hazards model based on all participants randomized to the RCT that compares survival time (weeks from randomization to relapse) across treatment groups. A subject’s survival time will be classified as censored at the point of study discontinuation (e.g., due to withdrawal of consent or due to a severe intervening non-psychiatric medical event) or at the end of follow-up, if relapse has not occurred. Kaplan Meier survival curves will be used for descriptive analyses of time to relapse. The Cox models will include treatment group and the three aforementioned stratification variables. Treatment groups will be compared on 5 variables at the time of randomization that have the potential to be associated with risk of relapse: anxiety, medical burden, executive dysfunction, psychomotor change, and treatment resistance prior to study entry; if the treatment groups differ significantly on one or more of these variables (p ≤ .05) and the variable in question is correlated with relapse (r > .30), the variable will be included as a covariate in the Cox model. The constant hazards assumption will be examined by examining the incremental contribution of a treatment x vulnerable period of relapse interaction (exploring the possibility of higher risk in the first 3 months following discontinuation).

#### Secondary hypotheses

H2 will be tested using separate mixed-effects linear regression analyses for weight, cholesterol, and triglycerides in the RCT. Subjects may be treated for glucose or lipid abnormalities that develop during the study. If a participant receives new treatment or a change of treatment for hyperlipidemia or hyperglycemia during the RCT, his/her pertinent metabolic data from that point onwards will be excluded from the mixed model, although the metabolic measures in question will continue to be collected for safety analyses and reporting to the Data Safety and Monitoring Board; based on data from STOP-PD, we expect that fewer than 5% of participants will have metabolic data censored for this reason. We chose not to include these post-randomization metabolic data as covariates in the mixed model, because doing so could confound ‘cause and effect’ and result in a biased estimate of the treatment effect, thereby compromising the primary goal of the analysis.

H3: Mixed-effects linear regression analysis will examine weight change from Acute Phase baseline.

Each statistical test for the primary and secondary hypotheses will involve a two-tailed alpha of 0.05.

#### Sample size determination and power analyses

##### Assumptions for power calculation for H1

We propose 20% as the minimal clinically meaningful difference in relapse rates between olanzapine and placebo over 36 weeks. A 20% difference would mean that 5 patients would need to be treated with olanzapine to prevent 1 case of relapse. Based on the review of literature [14-16,65] and STOP-PD stabilization data, we estimate that 15% of participants who are maintained on sertraline plus olanzapine will have a relapse in the RCT. A relapse rate of 35% in the sertraline plus placebo group would therefore be consistent with the hypothesized 20% difference between treatment groups. Based on our previous experience of conducting studies of the continuation and maintenance treatment of PD [12,65,66], we predict that attrition during the RCT will not exceed 10%. This attrition rate might appear to be low, but this is because those subjects most vulnerable to attrition will likely drop out in the phases prior to randomization. Moreover, data pertaining to H1 will be analyzed according to the intent to-treat principle, in that each subject will be classified in a treatment group based on randomized assignment without regard to medication adherence. Thus, if a participant chooses to discontinue one or both study medications in the RCT, every effort is made to continue research assessments for the entire course of randomized treatment or until relapse (whichever comes first). The proposed sample size will provide sufficient statistical power to detect clinically meaningful differences in H1 (Table 3).

**Table 3:** STOP-PD II: statistical power for survival analyses for hypothesis 1

**Frequency of relapse for sertraline + placebo**	**Frequency of relapse for sertraline + olanzapine**	**Attrition**	**Power**
40%	15%	10%	0.95
40%	15%	15%	0.94
35%	15%	10%	0.84
35%	15%	15%	0.82
35%	10%	10%	0.98
35%	10%	15%	0.97

##### Statistical power for H2

To account for attrition in H2, the power analyses assumed we would collect at least 8 of 15 repeated assessments of weight and 4 of 5 measures of total cholesterol and triglycerides during the RCT. The proposed design will have power ≥0.80 to detect differential slopes that result in standardized differences at endpoint ≥0.35 for weight and ≥0.40 for triglycerides and total cholesterol (Table 4). To put these in perspective, based on STOP-PD data, these effects correspond to group differences as small as 4.9 lbs in weight, 38 mg/dl in triglycerides, and 22 mg/dl in total cholesterol.

**Table 4:** STOP-PD II: statistical power of mixed-effects linear regression analyses for hypotheses 2 and 3

**Number of observations per subject**	**Standardized effect**	**ICC = 0.50 Lipids**	**ICC = 0.95 Weight**
4	0.35	0.74	0.77
4	0.40	0.84	0.85
4	0.45	0.89	0.93
8	0.35	-	0.80
8	0.40	-	0.89
8	0.45	-	0.95

##### Statistical power for H3

Power analyses for H3 assumed that we would collect at least 4 of the 6 planned assessments of weight during open-label treatment. The proposed design will have power >0.80 to detect differential slopes that result in a standardized difference as small as 0.40 by the end of the open-label phase (Table 4). To put this in context, of the participants in STOP-PD who completed 12 weeks of acute treatment, the younger group gained a mean 16.6 ± 16.5 lbs and the older group gained a mean 10.7 ± 11.3 lbs; the standardized effect size for this weight difference was 0.42.

## Discussion

### Antipsychotic discontinuation

Given the absence of data informing the continuation or discontinuation of antipsychotic medication in remitted PD, STOP-PD II provides the unique opportunity to study the effect of antipsychotic discontinuation (as opposed to switching antipsychotics) on antipsychotic-related weight gain and metabolic effects. It is unclear whether cessation of antipsychotic medication is associated with complete reversal of weight gain and metabolic effects, and if so how long this takes and whether there is an age effect.

### Accounting for depression-related weight change

PD can lead to considerable weight loss, in some cases 25% or more of ideal body weight. Analysis of antipsychotic-specific weight gain must account for depression-related weight loss and the subsequent restoration of pre-morbid weight as depression improves. Thus, we estimate weight prior to onset of the index depressive episode, using objective measures of premorbid weight (as opposed to self-report premorbid weight) whenever possible.

### Adult Age span approach

Studying adults across a wide age range has heuristic value, given that older age increases the risk of PD. In addition to the study’s secondary hypothesis and exploratory aim pertaining to an age effect on weight gain and metabolic changes, we will conduct exploratory analyses focused on other age-related questions, such as whether age effects the risk of relapse following antipsychotic discontinuation, and if so what are the clinical or biological variables that moderate or mediate this association.

### Measurement of insight

The Resolution of Delusions Scale (RODS) (unpublished data) was developed as a part of STOP-PD to assess a subject’s level of insight into his/her delusion. In STOP-PD II, we will explore whether lack of insight in to the false nature of a previously held, but now remitted, delusion increases the risk of relapse of PD, and if so whether this is mediated through neuropsychological processes.

### Pharmacogenetics

STOP-PD II will be the first study to examine genetic predictors of relapse and weight loss following discontinuation of antipsychotic medication. These findings have the potential to enhance the personalized care of patients with PD.

### Neuroimaging

Studies in schizophrenia suggest that antipsychotic medication may cause alterations in brain structure [67], but the observational nature of these studies is confounded by the potential effect of schizophrenia. The placebo-controlled antipsychotic discontinuation design of STOP-PD II offers the unique opportunity to study the specific effect of antipsychotic medication on brain structure. The investigators will conduct a supplemental study that will use magnetic resonance imaging during the RCT to compare the effects of olanzapine and placebo on brain structure and connectivity.

## Conclusion

STOP-PD II addresses an issue of profound clinical importance in the management of PD: what are the risks and benefits of continuing antipsychotic medication in remitted PD. The findings of this study will provide clinicians with a much-needed evidence base to guide the continuation treatment of one of the most disabling and lethal psychiatric disorders.

## Abbreviations

BMI: Body mass index; CGI: Clinical global impression scale; DAS: Delusion assessment scale; HAM-D: Hamilton depression rating scale; MMSE: Mini mental state examination; PD: Psychotic depression; RCT: Randomized controlled trial; SADS: Schedule of affective disorders and schizophrenia; SCID: Structured clinical interview for DSM; SSI: Scale for suicidal ideation; STOP-PD: Study of the pharmacotherapy of psychotic depression.

## Competing interest

Alastair Flint receives grant support from the NIMH, the Canadian Institutes of Health Research, and Lundbeck and has received honoraria from Janssen-Ortho, Lundbeck Canada, and Pfizer Canada.

Anthony Rothschild has received grant support from the National Institute of Mental Health (NIMH), Cyberonics, Takeda, and St. Jude Medical and has served as a consultant to Allergan, GlaxoSmithKline, Eli Lilly, Noven Pharmaceuticals, Pfizer, Shire Pharmaceuticals, and Sunovian.

Ellen Whyte has received research support from the NIMH, the National Institute of Child Health and Human Development (NICHD), the Department of Defense (DOD) and through a Small Business Innovation Research (SBIR) grant from Fox Learning Systems / National Institute of Neurological Disorders and Stroke (NINDS).

Benoit Mulsant currently receives research support from the Canadian Institutes of Health Research (CIHR), the US National Institute of Health (NIH), Bristol-Myers Squibb (medications for a NIH-funded clinical trial), and Pfizer (medications for a NIH-funded clinical trial). He directly own stocks of General Electric (less than $5,000). Within the past three years, he has also received some travel support from Roche.

Barnett Meyers receives research support from the NIMH. He is receiving medication donated by Pfizer and Eli Lilly for his NIMH trial. During the last three years he has provided legal consultation to AstraZeneca and research consultation for Forest Laboratories.

Matthew Rudorfer and Patricia Marino have no disclosures.

## Authors’ contributions

AJF, AJR, BHM, BSM, and EMW conceptualized and designed the study, contribute to the conduct of the study, and participate in the acquisition of data. PM and MVR contribute to the conduct of the study. AJF was responsible for drafting the manuscript. AJR, BMH, BSM, EMW, MVR, and PM reviewed the manuscript for intellectual content and contributed to its revision. All authors gave final approval of the version of the manuscript to be published.

## Pre-publication history

The pre-publication history for this paper can be accessed here:

http://www.biomedcentral.com/1471-244X/13/38/prepub
